# Enhancing medical Q&A systems with multimodal knowledge graphs and dual-layer attention mechanisms

**DOI:** 10.1371/journal.pone.0353112

**Published:** 2026-07-07

**Authors:** Guoqiang Qiu, Qingni Yuan, Yi Wang, Pengju Qu, Wei Jia

**Affiliations:** 1 Key Laboratory of Advanced Manufacturing Technology, Ministry of Education, Guizhou University, Guiyang, China; 2 The First People’s Hospital of Guiyang, Guiyang, China; 3 Engineering Training Center, Guizhou Institute of Technology, Guiyang, China; 4 College of Big Data and Information Engineering, Guizhou University, Guiyang, China; State Grid Corporation of China, CHINA

## Abstract

Medical intelligent question-answering (QA) systems have become important tools for improving the efficiency of healthcare services, and recent research has increasingly emphasized performance optimization and multimodal integration. However, existing systems still face several challenges in intent recognition, entity extraction, and multimodal knowledge fusion, particularly reduced accuracy in multi-label classification, heavy reliance on large-scale annotated data, and limited support for cross-modal retrieval. To address these issues, this study proposes a medical intelligent QA framework that integrates a dual-layer attention mechanism, a large language model, and a multimodal medical knowledge graph to improve system understanding and response generation in complex clinical scenarios. Specifically, we develop a text-based intent recognition model with a dual-layer attention architecture, in which a global contextual attention module is introduced to capture long-range semantic dependencies and improve multi-label classification performance. In addition, an instruction-tuned large language model is employed for zero-shot medical entity recognition, thereby reducing dependence on manually annotated datasets. Building on this foundation, we construct a multimodal medical knowledge graph comprising more than 15,000 associated medical images and develop a visualization-oriented retrieval interface using Flask and ECharts. Experimental results show that the proposed intent recognition model achieves a peak Micro-F1 of 94.42% on multiple benchmark datasets, outperforming several baseline methods. The LLM-based entity recognition module achieved competitive recall in medical entity extraction, demonstrating strong capability in identifying medical entities. User evaluation results further indicate that the system is effective and practical across a variety of medical query types. This study provides a feasible framework for advancing medical QA systems through improved intent recognition, low-resource entity extraction, and multimodal knowledge integration.

## 1. Introduction

Medical knowledge acquisition serves as a vital foundation for supporting clinical decision-making and enhancing diagnostic and treatment efficiency. Currently, there are three primary methods for acquiring medical knowledge. The first involves searching specialized medical databases, where users input medical keywords to obtain answers. However, keywords often fail to fully capture the query’s overall intent [1], limiting this method’s accuracy and adaptability to natural language questions. The second approach leverages general search engines or large language models (e.g., Google and DeepSeek) to directly process medical inquiries in natural language. Yet, their outputs typically lack structure, requiring manual filtering and verification, resulting in low efficiency. The third approach is based on traditional machine learning models and knowledge graph-based question-answering technology, typically comprising three core modules: named entity recognition, text classification (also known as text intent recognition), and knowledge graph construction. This method performs well in structured responses and semantic understanding, making it one of the most extensively researched and relatively mature technical paths currently.

However, the third approach still faces three key challenges: First, during text classification, although deep learning models perform well in most tasks, their accuracy often declines with increasing number of labels. This occurs because as the number of categories grows, models must distinguish between semantically similar categories with greater precision. Simultaneously, training data distribution across each category may become sparser, leading to overfitting or blurred classification boundaries. Maintaining or enhancing classification capabilities in multi-label scenarios remains an unresolved challenge. Second, in the named entity recognition (NER) stage, the deep learning methods employed are highly dependent on the quality of annotated data. Insufficient training data leads to poor model performance, while excessive data volume increases the risk of omissions or errors introduced by manual annotation, both of which adversely affect recognition accuracy. Furthermore, deep learning models typically require vast amounts of annotated data to effectively learn entity recognition tasks. In the medical field, however, acquiring large-scale, high-quality annotated data is costly and extremely time-consuming. Third, existing question-answering systems predominantly rely on Neo4j databases for storing graph data. However, Neo4j cannot directly display image data and struggles to seamlessly integrate the Q&A interface with graph visualization within a single system. This limitation hinders the effective combination of multimodal knowledge graphs and question-answering systems.

To address these challenges, this study proposes an intelligent medical Q&A framework that integrates dual-layer attention-based text classification, large language model-based entity recognition, and a knowledge graph enriched with associated medical images, thereby improving the integrated organization and presentation of medical knowledge within the system. Inspired by GCNet [2]’s use of global context attention to capture holistic image information in image recognition, this approach is adapted for text classification. By adding global contextual attention mechanisms before and after pooling operations, the model dynamically captures long-range dependencies and global semantic information within text, thereby enhancing its representation capabilities for complex textual features and classification performance. Subsequently, we construct an LLM-based medical entity recognition workflow by standardizing entity type definitions, designing precise instruction constraints, and aligning them with knowledge graph node labels, effectively resolving dataset dependency during the entity recognition phase. Finally, this paper constructs a local multimodal resource repository containing 15,183 medical images. A backend API server built on the Flask framework enables efficient interaction with the Neo4j graph database. Structured graph data is provided to the frontend via RESTful interfaces, and ECharts is leveraged to achieve integrated visualization of the multimodal knowledge graph and question-answering system.

## 2. Related work

### 2.1. Current state of text classification research

In text classification and intent recognition research, multimodal fusion, few-shot learning, and implicit intent understanding have become key focuses. For multimodal intent recognition, Li et al. [3] proposed a text-guided cross-modal attention mechanism to enhance non-textual modality features. Dong et al. [4] introduced a reinforcement learning-based multi-task framework to generate modality-relevant explanations and mitigate modality bias. Xia et al. [5] designed a method combining video feature enhancement with multimodal feature collaboration, achieving significant improvements on multimodal datasets. For context-aware intent recognition, Zhang et al. [6] combined machine reading comprehension with memory networks, utilizing self-attention and co-attention mechanisms for multi-turn dialogue intent recognition. Pan et al. [7] proposed a hybrid multi-intent recognition method for air-ground communication texts, modeling different text types separately. For small-sample and cross-lingual scenarios, Cao et al. [8] transformed cross-lingual intent recognition into masked language modeling tasks via prompt learning, significantly improving accuracy on low-resource languages. Zhang et al. [9] employed pre-trained language models and contrastive learning for gap-filling data augmentation, effectively mitigating data scarcity. For implicit intent recognition, Liu et al. [10] constructed the Chinese Implicit Intent Dataset (CIID) and pioneered prompt learning to uncover users’ true intentions. Despite progress across scenarios, existing methods still suffer from declining performance as label counts increase, hindering further development of multi-label classification techniques. Inspired by GCNet’s global context modeling in image recognition, this paper introduces a global attention mechanism to text classification tasks. By embedding global context attention modules before and after pooling operations, the model’s ability to capture long-range dependencies and global semantics is enhanced, significantly improving classification performance in multi-label text classification.

### 2.2. Current state of named entity recognition research

In current named entity recognition research, multiple studies have combined pre-trained language models with sequence annotation structures to enhance performance, addressing the specificity and complexity of domain-specific texts. For instance, in the field of traditional Chinese medicine, Yang et al.[11] proposed the BERT-BiLSTM-MHA-FUSION-CRF model, which enhances semantic representations through multi-head attention and multi-level fusion mechanisms, achieving significant accuracy improvements across multiple datasets. Zhu et al. [12] proposed ERPG, a prompt-guided generative approach for complex entities, effectively reducing invalid outputs and improving recognition quality. For medical texts, Song et al. [13] introduced semantic knowledge augmentation and global pointer optimization mechanisms, significantly improving nested entity recognition. Wang et al. [14] employed MCBERT combined with CNN-BiLSTM-CRF for dynamic semantic extraction, achieving an F1 score of 82.8% in electronic medical record recognition tasks. Li et al. [15] proposed a model integrating BERT with BiLSTM-CRF, incorporating part-of-speech and chunk analysis features, achieving an average F1 score of 89.45% across multiple biomedical datasets. Su et al. [16] introduced Global Pointer (GP), a novel fragment-based framework. Leveraging relative position information and a multiplicative attention mechanism, GP enhances fine-grained semantic capture and mitigates label imbalance through head-tail identification modules and a novel classification loss function. It achieves outstanding performance across multiple benchmark datasets while reducing parameters. He [17] and Qi [18] respectively enhanced local feature capture for Chinese medical and social media texts by introducing IDCNN and boundary expansion modules. Overall, current NER research is increasingly characterized by the deep integration of pre-trained models, multi-scale feature interaction, domain adaptation, and enhanced low-resource learning capabilities. Related efforts in other domains also reflect this trend toward data-efficient large-model learning under limited-data conditions. For example, Meng et al. proposed PowerMistral, a pre-trained large language model-based framework for few-shot wind power forecasting, further highlighting the broader potential of large models in data-scarce scenarios [19]. However, existing NER methods still rely heavily on large volumes of high-quality annotated data, and their performance is strongly affected by both data scale and annotation quality, while the annotation process itself remains costly and sensitive to noise. To address these limitations, this paper establishes a large-model-based medical entity recognition workflow by standardizing entity type definitions, designing precise instruction constraints, and aligning recognized entities with knowledge graph nodes. This workflow reduces dependence on manually annotated data and provides a flexible alternative for medical entity extraction in low-resource settings.

### 2.3. Research on intelligent question-answering mechanisms

In the research of intelligent question-answering mechanisms, the integrated application of knowledge graphs and large language models has become a key technological pathway for advancing intelligent services in specialized domains. Existing research indicates that related achievements have extensively permeated multiple vertical domains. In non-medical professional fields, system designs demonstrate deep adaptability to specific scenarios: Zhou et al. [20] combined large language models with facial recognition technology to construct a secure Q&A system capable of user identity perception, achieving intelligent access control interaction through local knowledge base retrieval and permission control; Li et al. [21] developed a specialized Q&A system for overseas COVID-19 case management using TF-IDF and Bi-LSTM+CRF models, enabling precise retrieval and visualization of pandemic-related knowledge; Li et al. [22] innovatively employed RAG frameworks and AI agent technology to address intangible cultural heritage preservation needs, establishing professional game analysis and Q&A mechanisms for data-scarce domains like Tibetan chess; Liu et al. [23] focused on geological exploration, constructing a mineral knowledge graph containing 22,000 entities based on the BERT model to achieve high-precision responses to natural language mineral queries. In the medical field, related research is particularly active, with Q&A system development emphasizing professionalism, safety, and personalized service capabilities. Zhou et al. [24] developed a medical knowledge graph Q&A system covering 14,000 diseases by combining multi-similarity matching algorithms with a Naive Bayes classifier; Guan et al. [25] proposed a joint mechanism for intent recognition and entity linking, significantly enhancing Q&A robustness under non-standard medical terminology; Qin et al. [26] integrated Neo4j knowledge graphs with large language models, employing LoRA fine-tuning to provide personalized Q&A services for diabetic patients; Sukhwal et al. [27] designed a long-form disease Q&A system leveraging LLM-KG synergy, effectively balancing medical factual accuracy with generative fluency; Hu et al. [28] employed a retrieval-based approach integrating Seq2Seq and TF-IDF models to construct a medical Q&A system; Wang et al. [29] proposed the knowledge-enhanced KEMedGPT model, focusing on medical consultation and pre-consultation scenarios; Shi et al. [30] utilized BERT and Naive Bayes methods to construct a medical knowledge graph and automated Q&A window. Although existing research has made significant progress in the accuracy and domain adaptability of professional Q&A systems, their underlying architectures still predominantly rely on graph databases like Neo4j as the core knowledge storage. However, Neo4j struggles to directly support image data storage and visualization integration, hindering the unified presentation and interaction of multimodal knowledge within Q&A interfaces. This limitation constrains the system’s ability to comprehensively express complex medical knowledge. To address this bottleneck, this paper constructs a local multimodal resource repository containing 15,183 medical images. A backend API service layer is built using the Flask framework to enable efficient interaction with Neo4j. Structured graph data is provided to the frontend via RESTful interfaces, and knowledge visualization is seamlessly integrated with Q&A functionality through ECharts. This approach provides a practical solution for integrating graph knowledge, associated images, and interactive display within the QA system.

## 3. Medical intelligent question-answering model

**Fig 1:**
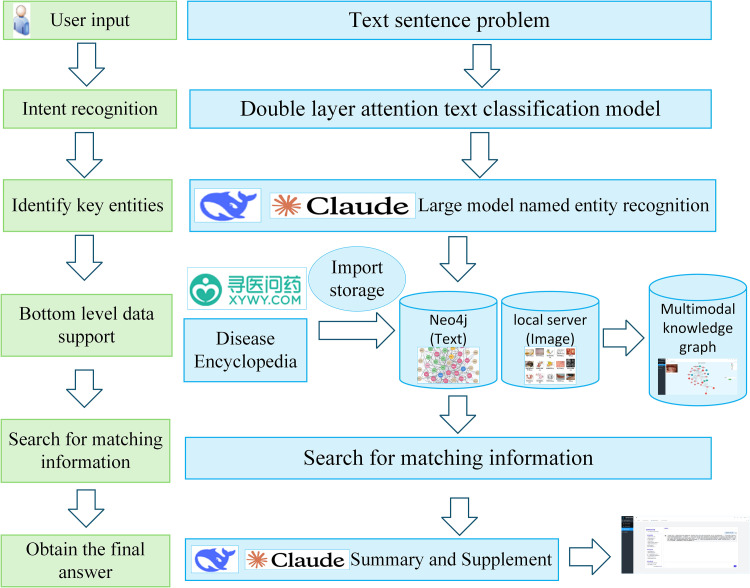
Overall workflow of the medical Q&A system.

This paper aims to construct an efficient and accurate medical intelligent question-answering model, as shown in Fig 1. This model adopts a layered design, forming a complete closed-loop from front-end interaction and back-end processing to underlying data storage. It encompasses user input processing, intent recognition, entity extraction, knowledge retrieval, answer generation, and visual presentation. Its core lies in integrating three key technologies: dual-layer attention-based text classification, large language model-based entity recognition, and multimodal knowledge graph retrieval. These components achieve efficient collaboration through a Flask backend service. Specifically, the input layer employs a dual-layer attention text classification model for intent recognition and dynamic matching of predefined response templates. Attention mechanisms focus on key semantic segments to enhance classification accuracy. Subsequently, large language models like Claude and DeepSeek perform medical entity recognition, precisely extracting keywords such as disease names, symptoms, and medications. Cypher templates optimize knowledge graph queries. Structured medical knowledge (including nodes like diseases, symptoms, medications, departments, and their relationships) stored in a Neo4j graph database, combined with associated images from a local image repository, enables multimodal text-image retrieval. Image path mapping and caching are managed via a local HTTP image server. Finally, retrieval results are populated into templates. After summarization and supplementation by large language models, multimodal answers are generated. These answers include textual descriptions, associated images, and knowledge graph visualization components, significantly enhancing user comprehension efficiency.

### 3.1. Intent recognition based on a dual-layer attention mechanism

**Fig 2:**
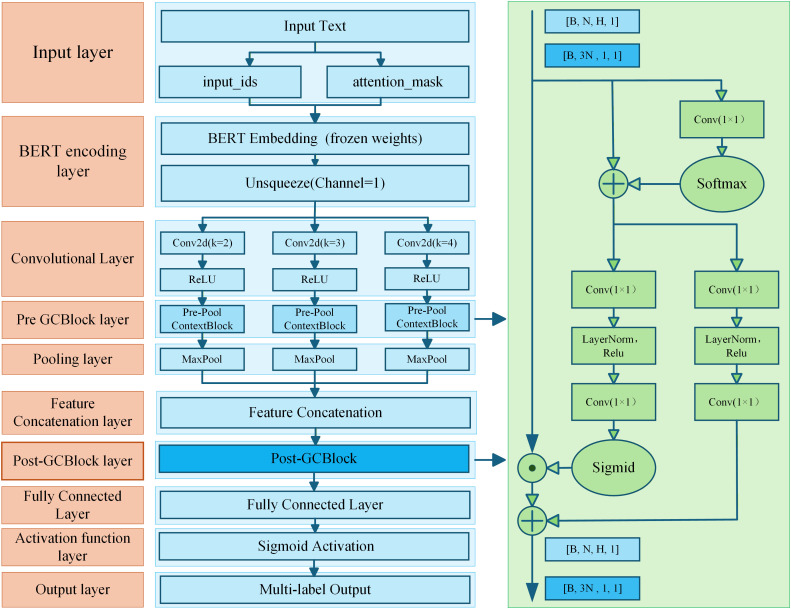
Dual-layer attention text classification model architecture.


To accurately understand user query intent, this paper designs a dual-layer attention text classification model. By incorporating multi-scale convolutions and a global context attention mechanism(GCNet), the model effectively enhances classification performance in multi-label scenarios. The overall operational flow of the model is illustrated in Fig 2: Given an input text sequence $X=\{x_{\text{1}},x_{\text{1}},\cdots x_{L}\}$={text}, where *L* denotes the sequence length, the sequence is first segmented into tokens and converted into a numerical tensor. Subsequently, each token is mapped to a context-aware vector *E* through the BERT pre-training model:

$$\begin{array}{c} E=BERT(X)\in R^{B\times L\times D} \end{array}$$ (1)

Here, B = 100 represents the batch size, and D = 768 denotes the dimension of the BERT hidden layer. Subsequently, the channel dimension is added, yielding the feature map $E^{\prime }\in R^{B\times L\times D\times \text{1}}$.

Subsequently, multi-scale convolutional feature extraction is performed on the feature maps by applying parallel convolutional operations using convolutional kernels of various sizes to the input features. For each feature map $F_{k}$ processed with a kernel size k∈{2,3,4}:


$$\begin{array}{c} F_{k}=ReLU(W_{k}*E^{\prime }+b_{k})\;\in R^{B\times N\times H\times \text{1}} \end{array}$$ (2)

Among these, ReLU is a nonlinear activation function that sets negative values to zero while preserving positive values, used to filter out irrelevant features and retain relevant ones. $W_{k}$ denotes the weight matrix of the convolutional kernel, * represents the convolution operation, $b_{k}$ is the bias vector of the convolutional kernel, *N* is the number of convolutional kernels, and *H* is the sequence length minus the kernel size, i.e., H= L−k.

Subsequently, the three feature maps $F_{k}$ were processed separately in the GCNet (Global Context Attention Mechanism Layer) designed in this paper. This layer mainly consists of three steps: spatial attention pooling, channel attention fusion, and feature fusion, as shown on the right of Fig 2:

For each feature map $F_{k}$, the attention weight matrix is first calculated through spatial attention mechanism:


$$\begin{array}{c} M=Softmax(W_{M}*F_{k})\;\in R^{B\times N\times H\times \text{1}} \end{array}$$ (3)

Among these, Softmax is a function that converts any numerical vector into a probability distribution, where $W_{M}\;\in R^{\text{1}\times N\times \text{1}\times \text{1}}$ is a 1 × 1 convolution kernel. Then, the weighted context vector *c* is computed:


$$\begin{array}{c} =\sum_{i=\text{1}}^{H}M[:,:,i,:]\cdot F_{k}[:,:,i,:]\in R^{B\times N\times \text{1}\times \text{1}} \end{array}$$ (4)

Among these,: denotes the fixed matrix’s corresponding positional dimension. Next, channel attention fusion is performed on *c*, which comprises two parallel branches: the channel multiplication branch:


$$\begin{array}{c} g_{mul}=\sigma (W_{{mul}_{\text{2}}}ReLU(W_{{mul}_{\text{1}}}\cdot c))\;\in R^{B\times N\times \text{1}\times \text{1}} \end{array}$$ (5)

Channel Addition Branch:


$$\begin{array}{c} g_{add}=W_{{add}_{\text{2}}}ReLU(W_{{add}_{\text{1}}}\cdot c)\;\in R^{B\times N\times \text{1}\times \text{1}} \end{array}$$ (6)

In the above branch computations, σ denotes the sigmoid activation function, while $W_{{mul}_{\text{1}}}$, $W_{{mul}_{\text{2}}}$, $W_{{add}_{\text{1}}}$, $W_{{add}_{\text{2}}}$ represent the feature matrices for each stage. The matrix $\overset{\prime }{\underset{k}{F}}$ is obtained by fusing the original features with the attention weights:


$$\begin{array}{c} \overset{\prime }{\underset{k}{F}}=F_{k}⊙g_{mul}⊕g_{add}\;\in R^{B\times N\times H\times \text{1}} \end{array}$$ (7)

Here, ⊙ and ⊕ denote element-wise multiplication and addition, respectively. The matrix $P_{k}$ is obtained by performing global max pooling on the features at each scale:


$$\begin{array}{c} P_{k}={max}_{i}\;\overset{\prime }{\underset{k}{F}}[:,:,i,:]\;\in R^{B\times N} \end{array}$$ (8)

Concatenate the pooling results from all scales into the matrix *P*:


$$\begin{array}{c} P=concat[p_{\text{1}},p_{\text{2}},p_{\text{3}}]\;\in R^{B\times \text{3}N} \end{array}$$ (9)

The concatenated features are reshaped into a 4-channel tensor $P^{\prime }\in R^{B\times \text{3}N\times \text{1}\times \text{1}}$. This tensor passes through the next layer’s global context attention mechanism, calculated using Equations (3)–(7), ultimately yielding $P^{\prime \prime }\in R^{B\times \text{3}N\times \text{1}\times \text{1}}$. The processed features $P^{\prime \prime }\;$undergo classification via a fully connected layer:


$$\begin{array}{c} y=\;\sigma (W_{linear}\cdot P^{\prime \prime }+b_{linear})\;\in R^{B\times n} \end{array}$$ (10)

Here, *n* denotes the number of text categories, *y* represents the final probability distribution matrix across the n text categories, while $W_{linear}$ and $b_{linear}$ denote the feature matrix and bias of the fully connected layer.

### 3.2. Large language model-based named entity recognition

To accurately extract medical entities from user queries while reducing reliance on labeled data, this study employs large language models (LLMs) such as DeepSeek and Claude for named entity recognition (NER) through prompt-based extraction. This approach achieves efficient model transfer without additional training by constraining model outputs via carefully designed prompts. The workflow proceeds as follows: First, access the selected LLM service via the appropriate API. Next, define the entity types and their semantic scope based on the specific requirements of the medical question-answering task. For example, set the entity type set format as: entity_types = [‘disease’, # Disease: heart disease, cold, etc.]. This definition must align with existing entity types in the underlying medical knowledge graph to ensure effective mapping between identified entities and graph content. Next, provide explicit instruction constraints to the LLM, such as requiring: “1. Do not omit entities that appear repeatedly in the text; 2. Maintain the integrity of professional terminology.” Finally, input the text to be identified {text} and the specified output format into the model. Through these instructional controls and format constraints, relevant entity information can be extracted from user questions in a structured manner, while reducing dependence on large-scale annotated data.

Algorithm: Large Language Model Named Entity Recognition

**Input:** User text *{text}*

**Output:** Predicted entities in list format

1: Initialize environment and API configuration

2: Define entity type set *entity_types*

3: Construct the prompt variable *prompt* using the user input *{text}*

4: while retry count < 3 do:

5:   Call the selected LLM API to obtain a response

6:   Parse response text to extract entities

7:   if success then break

8: Format output as nested list: [

   [‘entity_type’, ‘entity_text’],

   [‘entity_type’, ‘entity_text’],

   ...]

### 3.3. Question-answering mechanism based on multimodal medical knowledge graphs

**Fig 3:**
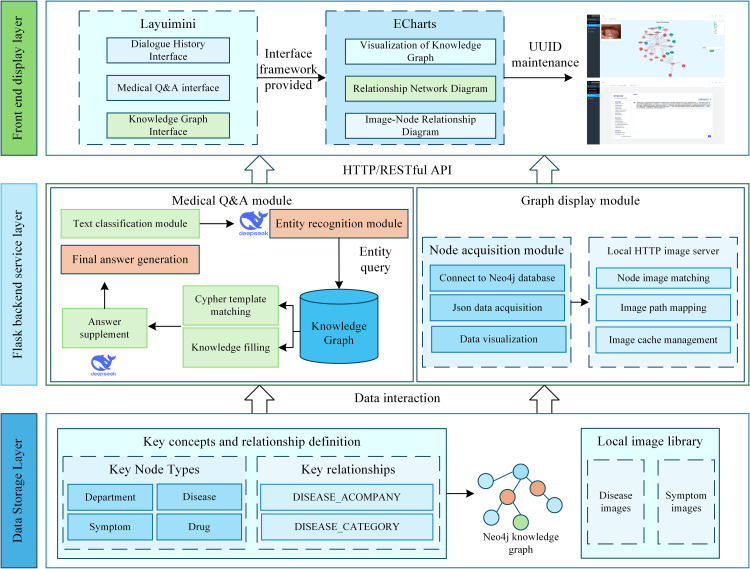
Architecture of a question-answering mechanism for multimodal medical knowledge graphs.

To apply the aforementioned intent recognition and entity recognition modules to practical scenarios, this section constructs a comprehensive medical intelligent question-answering mechanism. Its overall architecture, as shown in Fig 3, primarily comprises three major components: multimodal knowledge base construction, backend service logic, and frontend visual interaction.

This paper constructs a multimodal knowledge base based on Neo4j, whose structure is depicted in the data storage layer at the bottom of Fig 3. This knowledge base contains entities labeled with terms such as “Disease,” along with relationships labeled as “DISEASE_ACOMPANY” (complications), while also integrating a local resource repository containing 15,183 medical images. By establishing a local HTTP server, the system achieves effective mapping between image storage paths and knowledge graph nodes, enabling multimodal information association and access.

The backend service is built using the Flask framework and provides external services via RESTful APIs. As shown in the middle section of Fig 3, its core workflow proceeds as follows. First, upon receiving a user query, the system invokes the intent recognition model described in Section 3.1 to parse the query intent. Subsequently, the entity recognition module described in Section 3.2 is used to extract key medical entities. The recognized intent and entities are then combined to generate Cypher query statements for retrieving relevant information from the Neo4j knowledge graph and local image repository. Finally, the system populates the retrieval results into a predefined response template and invokes a large language model (LLM) to summarize and refine the content, generating a final answer with a clear structure and fluent language.

The front-end visualization layer employs a combination of Layuimini and ECharts technologies. As shown in the upper part of Fig 3, the Layuimini framework handles overall layout and interactive interface construction, while ECharts specializes in rendering complex relational structures like knowledge graphs. Together, they integrate text, image, and graph data returned from the backend into a unified view, ultimately providing users with an intuitive and fluid interactive experience maintained via UUIDs.

### 3.4. Ethics statement

The end-to-end user evaluation in Experiment 3 involved five domain experts and twelve general participants recruited from 13 March 2026 to 17 March 2026. Ethical review exemption for this study was granted by the Medical Ethics Committee of the First People’s Hospital of Guiyang. Written informed consent was obtained from all participants prior to the evaluation. No minors were involved in the study. The general participants were anonymous, and no personally identifiable information was collected or retained.

## 4. Experiment

To evaluate the proposed method, three experiments were designed at two complementary levels: system engineering validation and system-level validation with expert and user evaluation.

System Engineering Validation: Experiments 1 and 2 assess the effectiveness of the core technical modules. Experiment 1 examines whether the convolutional layer design is appropriate for medical text classification and whether the dual-layer attention mechanism improves classification performance. Experiment 2 evaluates the performance of large language models in medical named entity recognition by comparing them with conventional methods.

System-Level Validation with Expert and User Evaluation: Experiment 3 evaluates the integrated medical QA system as a whole in representative clinical question scenarios. Both medical experts and general users assess the system responses across four dimensions, including correctness, completeness, safety, and question-answer matching. The expert group provides a professional assessment of the medical quality and reliability of the generated answers, whereas the general-user group reflects user-perceived quality and practical acceptability. Across 16 types of clinical questions, this experiment is intended to validate the end-to-end performance, response quality, and practical applicability of the integrated system as a medical QA tool.

### 4.1. Experimental preparation

#### 4.1.1. Dataset.

The experiment selected four datasets. In Experiment 1: Text Classification Model Ablation Study, this paper utilized a self-built medical text classification dataset MTCD (Medical Text Classification Dataset) alongside two public datasets: Weibo and Toutiao (Weibo: https://github.com/SophonPlus/ChineseNlpCorpus. Toutiao: https://github.com/aceimnorstuvwxz/toutiao-multilevel-text-classfication-dataset/tree/master). In Experiment 2: Large Model Entity Recognition Evaluation, this study employed a public medical entity recognition dataset CMEDQANER (CMEDQANER: https://github.com/alibaba-research/ChineseBLUE) containing 11 entity labels including diseases, symptoms, and medications. When constructing the MTCD dataset, we first identified 16 distinct question-answering types based on features extracted from structured data crawled from the Xunyiwenyao website. Subsequently, we collected user-submitted question texts from open Chinese medical information websites and public medical Q&A datasets, then filtered and cleaned the data. Using the extracted textual features, we provided corresponding question-answering templates to the large language model for data augmentation, ultimately generating the MTCD dataset. Specific statistical information for the MTCD dataset is shown in Table 1, where symbols like [Disease] denote entity slots. The entire annotated dataset was divided into a test set and a training set in a 2:8 ratio, comprising a total of 27,582 samples. The Weibo dataset consists of sentiment-labeled sentences collected from Sina Weibo, with approximately 50,000 positive and negative comments each. The Toutiao dataset comprises 382,688 sentences collected from the Toutiao client, categorized under 15 news tags including automotive, agriculture, and sports. The self-constructed dataset used in this study was compiled from publicly accessible online sources, including structured disease pages from the Xunyiwenyao website, open Chinese medical information websites, and public medical question-answering datasets. Only content that was publicly accessible at the time of collection was used, and no login-protected, paywalled, or access-restricted material was collected. Data collection and subsequent analysis were conducted in accordance with the applicable terms and conditions of the original data sources. Because some raw webpage content and associated medical images may remain subject to copyright or website-use restrictions, these original materials are not redistributed in this study. Instead, we provide the processed knowledge graph data, implementation code, and related derived resources, and refer readers to the original public sources for access to the underlying raw materials.

**Table 1:** MTCD text classification dataset statistics.

Label ID	Labels	Example	Train+test	Total
1	Disease Definition	What is [Disease]?	963 + 254	1217
2	Disease Causes	What causes [Disease]?	1688 + 415	2103
3	Disease Symptoms	What are the symptoms of [Disease]?	1828 + 466	2294
4	Symptoms Check Disease	What disease does [Symptom] indicate?	738 + 192	930
5	Treatment Methods	What is the treatment for [Disease] to recover?	3027 + 758	3785
6	Department	Which department should I see for [Disease]?	1365 + 369	1734
7	Prevention	How can [Disease] be prevented?	1397 + 344	1741
8	Contraindications	Are there any dietary restrictions for patients with [Disease]?	1130 + 265	1395
9	Inspection Plan	What specific tests are needed to diagnose [Disease]?	1089 + 255	1344
10	Cure Rate	What is the cure rate for [Disease]?	1447 + 369	1816
11	Complications	What other diseases can [Disease] cause?	1550 + 362	1912
12	Drug Applicability	What diseases is [Drug] used for?	1176 + 298	1474
13	Treatment Cost	What is the approximate cost of treating [Disease]?	1143 + 278	1421
14	Treatment Duration	How long does the treatment for [Disease] last?	1302 + 324	1626
15	Contagiousness	Is [Disease] contagious?	943 + 225	1168
16	Incidence Rate	Is [Disease] common?	1290 + 332	1622
	**Total**	16	22076 + 5506	27582

#### 4.1.2. Training environment.

The model implementation was programmed in Python within a CUDA environment. We trained the model by running the program on a single NVIDIA 4090 24GB GPU. The system environment was Windows 11, utilizing the PyTorch framework version torch1.11.0 + cu113. Tables 2 and 3 list the experimental hardware and software configurations, respectively.

**Table 2:** Experimental hardware configuration.

Hardware and Systems	Configuration
CPU	i9-12900KS
Memory	64GB
Graphics card	NVIDIA 4090 24GB
Hard disk	1T
Systems	Windows11

**Table 3:** Experimental software environment.

Software	Configuration
Anaconda	4.12.0
PyTorch	torch1.11.0 + cu113
Python	Python3.9

#### 4.1.3. Parameter settings and evaluation metrics.

In Experiment 1: Text Classification Model Ablation Study, the pre-trained BERT-wwm model was employed to encode text data during the experimental phase. Subsequently, features were extracted from the encoded text using a convolutional layer and a dual-layer attention layer, followed by text classification. The model’s key parameters were configured as specified in Table 4, with their respective values explicitly defined.

**Table 4:** Model parameter setting.

Parameter	Value
Batch size	100
Epoch	100
Learning rate	5e-6
Max length	128
Number of kernels	256
Embedding dim	768

In the experimental setup, each training batch comprised 100 samples, and the learning rate was set to 5e-6. For the BERT-wwm encoder, the maximum input sequence length was set to 128, while the hidden size was 768. In the TextCNN module, the number of convolutional kernels was 256. The model was trained for 100 epochs.

This study employs precision (*P*), recall (*R*), and F1-score (*F*1) as the basic evaluation metrics for model performance [31–33], as shown in the following equation:


$$\begin{array}{c} P=\frac{TP}{TP+FP} \end{array}$$ (11)

$$\begin{array}{c} R=\frac{TP}{TP+FN} \end{array}$$ (12)

$$\begin{array}{c} F\text{1}=\frac{\text{2}\times P\times R}{P+R} \end{array}$$ (13)

Among these, *TP* (True Positive) refers to the number of samples correctly predicted as positive by the model, *FP* (False Positive) refers to the number of samples incorrectly predicted as positive by the model, and *FN* (False Negative) refers to the number of samples incorrectly predicted as negative by the model.

In Experiment 1, since the task is a multi-class text classification problem, **accuracy (A)** was further introduced as a supplementary evaluation metric [34]:


$$\begin{array}{c} A=\frac{TP+TN}{TP+TN+FP+FN} \end{array}$$ (14)

Where TN (True Negative) denotes the number of samples correctly predicted as negative. In addition, Micro−F1, Macro-F1, and Weighted-F1 were adopted to provide a more comprehensive evaluation of multi-class classification performance. Let $F{\text{1}}_{i}$ denote the F1-score of the *i*-th class, *C* the total number of classes, $n_{i}$ the number of samples in the *i*-th class, and $N=\sum_{i=\text{1}}^{C}n_{i}\;$the total number of samples. Then:


$$\begin{array}{c} Micro-F\text{1}=\frac{\text{2}\times P_{micro}{\times R}_{micro}}{P_{micro}+R_{micro}} \end{array}$$ (15)

$$\begin{array}{c} P_{micro}=\frac{\sum_{i=\text{1}}^{C}TP_{i}}{\sum_{i=\text{1}}^{C}\left(TP_{i}+FP_{i}\right)},\;R_{micro}=\frac{\sum_{i=\text{1}}^{C}TP_{i}}{\sum_{i=\text{1}}^{C}\left(TP_{i}+FN_{i}\right)} \end{array}$$ (16)

$$\begin{array}{c} Macro-F\text{1}=\frac{\text{1}}{C}\sum_{i=\text{1}}^{C}{F\text{1}}_{i} \end{array}$$ (17)

$$\begin{array}{c} Weighted-F\text{1}=\sum_{i=\text{1}}^{C}{\frac{n_{i}}{N}F\text{1}}_{i} \end{array}$$ (18)

Among these metrics, Micro−F1 reflects the overall classification performance over all samples, Macro−F1 emphasizes balanced performance across classes, and Weighted−F1 takes class distribution into account, making it more suitable for imbalanced multi-class datasets.

In Experiment 2, which focuses on medical named entity recognition, model performance was mainly evaluated using precision, recall, and F1-score, since these metrics are the most commonly used and sufficient indicators for sequence labeling tasks.

### 4.2. Experiment 1: Text classification model ablation study

To evaluate the classification performance of the proposed dual-layer attention text classification model, we first replaced the TextCNN [35] layer in the baseline model with several alternative text encoding structures, including TextRNN, DPCNN [36], DPRNN [37], and FastText [38], and conducted comparative experiments on the MTCD dataset. The results are shown in Table 5. Overall, among the compared backbone structures, the baseline model using TextCNN achieved relatively strong and stable performance, supporting its use as the baseline encoder in this study. On this basis, after introducing the proposed dual-layer attention mechanism, the model achieved the best performance among the compared methods in terms of Accuracy, Recall, Micro-F1, Macro-F1, and Weighted-F1. These results indicate that the proposed attention structure can effectively improve the overall classification performance on the self-built medical text classification dataset.

**Table 5:** Comparison of classification performance on the MTCD dataset.

Model	A	P	R	Micro-F1	Macro-F1	Weighted-F1
TextRNN	74.71	82.22	71.39	74.71	69.42	71.26
FastText	80.27	89.07	77.84	80.27	80.61	80.19
DPCNN	91.33	93.06	91.63	91.33	92.10	91.32
DPRNN	84.32	87.79	83.03	84.32	81.93	82.63
TextCNN(Base)	93.64	**94.79**	94.27	93.64	94.47	93.66
Ours	**93.93**	94.70	**94.76**	**93.93**	**94.72**	**93.92**

A, P, R, Micro-F1, Macro-F1, and Weighted-F1 are reported as percentages (%).

To further analyze the performance of the proposed model across different categories, Table 6 presents the fine-grained classification results for 16 labels, including the support for each category, as well as the numbers of correctly and incorrectly classified samples. For simplicity, the label categories are represented by Label ID throughout the manuscript, with the corresponding label names provided in Table 1; the same notation is also adopted in Tables 15–17. The results indicate that the proposed model achieves high classification performance in most categories, but it still encounters difficulties in distinguishing between a few semantically similar labels, such as Disease Definition, Disease Causes, and Disease Symptoms, which is reflected in relatively low Precision, Recall, and F1 values.

**Table 6:** Class-wise performance of the proposed model on the MTCD dataset.

Label ID	P	R	F1	Support	Correct	Wrong
1	86.07	82.68	84.34	254	210	44
2	82.82	84.82	83.81	415	352	63
3	81.22	80.17	80.69	464	372	92
4	98.45	99.48	98.96	192	191	1
5	92.44	91.95	92.20	758	697	61
6	99.73	99.73	99.73	369	368	1
7	99.42	99.13	99.27	344	341	3
8	97.32	95.85	96.58	265	254	11
9	96.89	97.65	97.27	255	249	6
10	94.75	97.83	96.27	369	361	8
11	96.78	91.44	94.03	362	331	31
12	93.55	97.32	95.39	298	290	8
13	99.28	99.64	99.46	278	277	1
14	99.08	99.69	99.38	324	323	1
15	98.24	99.11	98.67	225	223	2
16	99.10	99.70	99.40	332	331	1

The main confusion scenarios are summarized in Table 7. It can be observed that misclassifications primarily occur between semantically adjacent categories, such as disease symptoms, disease causes, treatment methods, and complications. Taking symptom-related questions as an example, their expressions often contain similar lexical patterns to those of cause-related or treatment-related questions, making them more prone to confusion. This indicates that the remaining classification errors primarily stem from semantic overlaps between similar medical question-answering types, rather than a lack of overall classification ability in the model.

**Table 7:** Major confusion cases of the proposed model on the MTCD dataset.

True Label ID	Pred. Label ID	Count	Confusion Rate	Brief Description
3	2	46	9.91%	Symptoms and causes overlap most.
2	3	22	5.30%	Cause queries often include symptoms.
5	3	21	2.77%	Treatment queries mix in symptoms.
1	3	19	7.48%	Definitions often contain symptom-like expressions.
11	3	17	4.70%	Complications are often expressed as symptoms.
3	5	16	3.45%	Symptoms overlap with treatment questions.

To further verify the robustness of the dataset construction strategy and the generalization ability of the model, this paper also conducted supplementary experiments on MTCD-n (i.e., the self-built dataset after removing all LLM-based template augmentation) as well as the public datasets Weibo and Toutiao. The results are presented in Table 8. On MTCD-n, our model still outperforms the baseline model across all evaluation metrics, indicating that the observed performance gains do not solely rely on template-based data augmentation. This finding suggests that the model is able to capture semantically meaningful intent-related features from the original data distribution, rather than merely fitting to artificially constructed template patterns. Furthermore, on the two public datasets Weibo and Toutiao, which contain naturally occurring open-domain texts with different linguistic styles and label distributions, our model also consistently outperforms the baseline methods. These results demonstrate that the proposed method is not over-aligned with the predefined QA-like structures introduced during augmentation, but instead maintains strong transferability and robustness across both medical and general-purpose text classification tasks.

**Table 8:** Comparison of classification performance on MTCD-n and public datasets.

Dataset	Model	A	P	R	Micro-F1	Macro-F1	Weighted-F1
MTCD-n	TextCNN(Base)	75.69	84.54	74.40	75.69	78.23	75.57
MTCD-n	Ours	**79.64**	**85.07**	**84.45**	**79.64**	**84.67**	**79.72**
Weibo	TextCNN(Base)	92.25	92.25	92.25	92.25	92.25	92.25
Weibo	Ours	**94.42**	**94.41**	**94.42**	**94.42**	**94.42**	**94.42**
Toutiao	TextCNN(Base)	87.39	81.09	80.97	87.39	81.00	87.35
Toutiao	Ours	**88.46**	**82.03**	**82.07**	**88.46**	**82.04**	**88.43**

### 4.3. Experiment 2: LLM-based entity recognition evaluation

To evaluate the entity recognition performance of large language models, the specific experimental methodology is as follows: by applying character-length filtering and random sampling to the original CMEDQA dataset, 300 samples ranging from 15 to 50 characters in length were obtained. This process excluded excessively short or long sentences while ensuring sufficient randomness. For the large language model-based methods, each experiment was repeated three times under the same setting, and the mean values together with standard deviations were reported to improve the reliability of the evaluation. The entity recognition task was then performed using the large language models via the method described in Section 3.2.

As shown in Table 9, the first five cases represent the CMedQANER test results of traditional deep learning models from [15] and [16], while the last four cases show the results of different large language models. Traditional deep learning models still outperform large language models in precision and overall F1. However, some large language models achieve competitive recall, and claude-sonnet-4–6 attains the highest recall (81.49% ± 0.73%). In terms of processing time, deepseek-v3.2 is the fastest among the tested large language models (243.12 ± 2.67 s). Overall, some large language models achieve relatively high recall in this task, but their substantially lower precision and F1 indicate that they are not yet competitive with the strongest traditional baselines in overall NER performance. The relatively small standard deviations also indicate that the repeated runs are reasonably stable.

**Table 9:** Performance comparison of different NER methods on the CMEDQA dataset.

Model	P(± SD)	R(± SD)	F1(± SD)	T(s)(± SD)
GP(BERT) [16]	76.20	79.94	77.98	/
GP(ALBERT) [16]	77.35	71.04	73.98	/
GP(ERNIE) [16]	70.42	71.24	70.63	/
GP(RoBERTa) [16]	78.73	79.40	**79.01**	/
BiLSTM+CRF [15]	**82.79**	77.19	78.76	/
claude-sonnet-4–6	41.18 ± 0. 33	**81.49** **±** **0. 73**	54.71 ± 0.46	847.6 ± 72.41
deepseek-v3.2	36.80 ± 0.30	70.54 ± 0. 72	48.36 ± 0.43	**243.12** **± 2.67**
gemini-2.5-pro	39.86 ± 0.20	46.21 ± 0.34	42.80 ± 0.22	645.97 ± 6.57
gpt-5.2-chat	34.79 ± 0.26	78.76 ± 1.11	48.26 ± 0.41	648.08 ± 24.23

As shown in Table 10, a temperature sensitivity analysis was further conducted on claude-sonnet-4–6, which showed relatively strong performance in the NER task. The results indicate that the model performance remains relatively stable under different temperature settings, with only slight variations in F1 score. Among these settings, temperature = 0.8 achieves the best overall performance, with the highest F1 (55.22% ± 0.15%) and recall (83.06% ± 0.20%).

**Table 10:** Temperature sensitivity analysis of Claude-Sonnet-4-6 for NER.

Temperature	P ± SD	R ± SD	F1 ± SD	T(s) ± SD
0	41.18 ± 0.33	81.49 ± 0.73	54.71 ± 0.46	847.6 ± 72.41
0.2	40.92 ± 0.09	81.92 ± 0.17	54.58 ± 0.12	**800.91 ± 44.09**
0.5	**41.44 ± 0.32**	78.01 ± 5.22	54.05 ± 1.04	1344.10 ± 171.28
0.8	41.36 ± 0.16	**83.06 ± 0.20**	**55.22 ± 0.15**	929.08 ± 89.20

As shown in Table 11, we further report the entity-type-wise performance of claude-sonnet-4–6 under its best-performing setting, namely temperature = 0.8. The results show clear differences across entity types. The model performs relatively well on crowd, disease, body, and drug, while weaker results are observed for time, feature, test, and physiology. In addition, the comparison between the real and predicted counts suggests that the model tends to over-identify some entity types, especially time, feature, physiology, and symptom. These results indicate that the method is more effective for entity categories with clearer semantic boundaries, while fine-grained or ambiguous categories remain more challenging.

**Table 11:** Entity-type-wise NER performance of Claude-Sonnet-4-6 at temperature 0.8.

Entity Type	P ± SD	R ± SD	F1 ± SD	Real	Predict ± SD
disease	66.13 ± 0.42	71.61 ± 0.81	68.6 ± 0.57	268	289.67 ± 2.62
symptom	38.54 ± 0.72	81.05 ± 0.86	52.24 ± 0.81	215	456.67 ± 6.80
drug	52.39 ± 2.33	66.67 ± 2.05	58.62 ± 1.46	26	33.33 ± 1.70
body	49.37 ± 0.17	80.82 ± 0.00	61.30 ± 0.13	171	284.00 ± 3.56
treatment	29.08 ± 0.84	68.08 ± 1.33	40.74 ± 0.93	83	188.33 ± 6.12
test	19.91 ± 0.25	38.46 ± 0.00	26.24 ± 0.22	48	84.67 ± 0.47
crowd	60.80 ± 0.35	89.41 ± 0.96	72.38 ± 0.43	96	141.67 ± 1.70
time	6.58 ± 0.33	63.64 ± 3.71	11.93 ± 0.60	24	227.33 ± 2.36
physiology	22.64 ± 0.52	74.75 ± 1.43	34.75 ± 0.71	37	116.33 ± 3.30
feature	8.40 ± 0.49	48.72 ± 3.63	14.33 ± 0.87	13	78.67 ± 1.70
department	28.69 ± 1.02	83.33 ± 3.37	42.68 ± 1.52	28	49.00 ± 0.00

### 4.4. Experiment 3: System-level validation with expert and user evaluation

**Fig 4:**
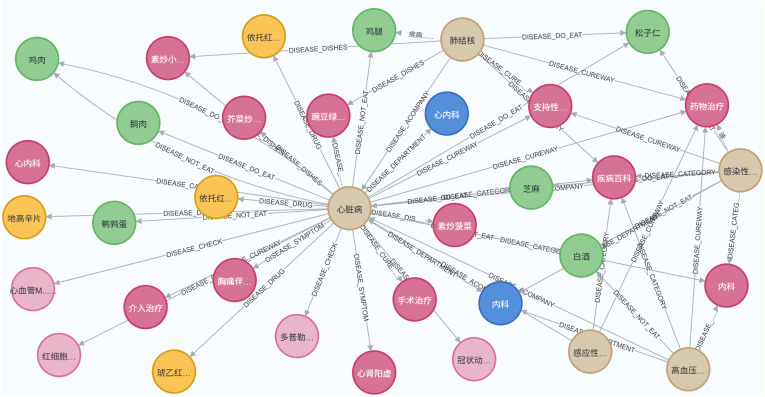
Medical knowledge graph stored on Neo4j (Partial).

**Fig 5:**
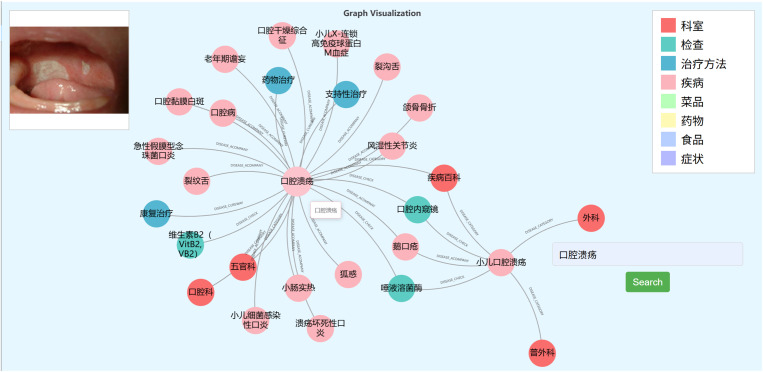
Multimodal medical knowledge graph based on ECharts (Partial).

**Fig 6:**
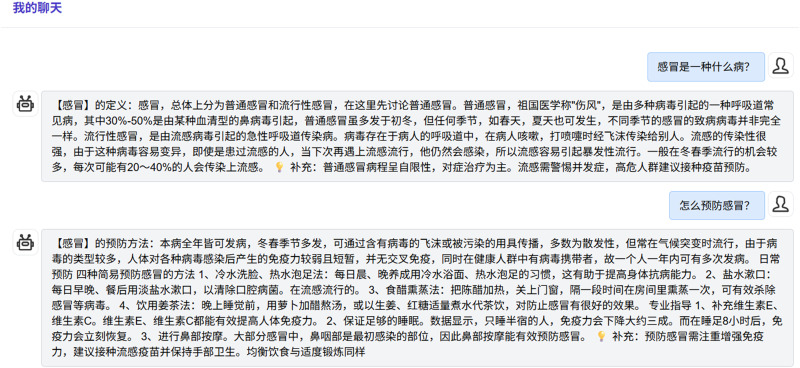
Medical knowledge graph Q&A system Q&A Example.

The number of medical nodes and relationships crawled from the disease encyclopedia section of Xunyiwenyao.com (Seek Medical Advice Network: https://www.xywy.com/) is shown in Tables 12 and 13, comprising 9 node types and 10 relationship types. Partial knowledge graphs constructed in the Neo4j database based on these data are illustrated in Fig 4. Selected multimodal knowledge graphs visualized in the ECharts frontend interface are shown in Fig 5. Fig 6 presents example Q&A interactions using cold definition and prevention as representative cases.

**Table 12:** Medical entity statistics.

No.	Entity	Number of entities
1	Category	55
2	Check	3353
3	Cureway	544
4	Department	54
5	Disease	4506
6	Dishes	8808
7	Drug	3828
8	Food	366
9	Symptom	5998
	Total	27512

**Table 13:** Statistical information on inter-healthcare entity connections.

No.	Relationship	Number of relationships
1	DISEASE_ACOMPANY	12024
2	DISEASE_CATEGORY	25587
3	DISEASE_CHECK	39418
4	DISEASE_CUREWAY	21047
5	DISEASE_DEPARTMENT	16781
6	DISEASE_DISHES	40221
7	DISEASE_DO_EAT	22230
8	DISEASE_DRUG	59736
9	DISEASE_NOT_EAT	22239
10	DISEASE_SYMPTOM	54710
	Total	313993

The medical text and image resources used for the multimodal knowledge graph were derived from publicly accessible third-party medical websites; details of data-source compliance and redistribution restrictions are described in the Dataset section.

Since the construction of the knowledge graph in this paper primarily relies on structured and semi-structured web page fields, rather than open semantic extraction based on free text, data noise control mainly focuses on the structured processing flow, including web page parsing result verification, null and outlier filtering, duplicate record removal, field mapping consistency check, entity naming normalization, and schema consistency check.

For image node assignment, this study achieves automatic alignment between image resources and knowledge graph nodes based on the original correspondence between entity pages and display images in the source webpages, combined with entity names and image file naming conventions. During the construction process, we eliminated samples with inconsistent names, abnormal naming, missing information, or entries that could not be uniquely matched, thereby improving the reliability of multimodal node mapping.

To ensure the usability of the knowledge graph, this paper further conducted three aspects of quality inspection after the graph construction was completed. Firstly, normalized processing and similar node screening were performed on entity names of the same type to reduce potential duplications caused by differences in naming granularity, alias expressions, or field redundancy. The inspection results indicated that most of the high-similarity nodes identified belonged to differences in concept hierarchy, subdivision of departments, or different expression granularity, rather than strictly defined duplicate entities. Secondly, focusing on the design of the graph structure, a pattern consistency check was conducted on the triples, with a focus on verifying whether the head entity type, relationship type, and tail entity type conform to predefined schema constraints. Finally, 100 triples were extracted using uniform sampling based on relationship labels, and experts in the medical field were invited to verify them from the perspectives of pattern consistency and content rationality. The results showed that most of the sampled triples were basically reasonable in terms of pattern and content, with a small number of samples (19 in total) having doubts that required further verification. These doubts mainly concentrated on diet/dish-related relationships, as well as a small number of drug and complication-related relationship types. The issues were more reflected in the instability of the semantic boundaries of relationships or insufficient medical specificity, rather than a loss of control over the overall entity type system of the graph. Overall, the constructed graph can better support the knowledge retrieval and answer generation requirements of this paper’s question-answering system, but there is still room for further optimization in some weakly structured relationships.

To more comprehensively evaluate the overall performance of the medical question-answering system in real question-answering scenarios, this study designed an end-to-end user evaluation involving both domain experts and general participants. For each intent category, three representative questions were selected for assessment, and the mean scores together with standard deviations were calculated based on the participants’ ratings. Table 14 presents the scoring criteria used in this evaluation. The criteria were developed through discussions with domain experts. System responses were assessed from four dimensions, namely Correctness, Completeness, Safety, and Match, with each dimension scored on a 2-point scale. This design provides a more intuitive reflection of answer quality in realistic QA scenarios and supports a more structured end-to-end evaluation of system performance [39].

**Table 14:** Scoring criteria for the end-to-end user evaluation.

Criterion	2	1	0
Correctness	Accurately addresses the core medical issue	Generally correct, with minor inaccuracies	Contains a clear medical error
Completeness	Covers the key information with no major omissions	Covers some key points but is incomplete	Omits important information
Safety	No significant safety concerns identified	Low risk, but important warnings are missing	Contains advice that may mislead patients or pose risk
Relevance	Directly addresses the main point of the question	Mostly relevant, but slightly deviates from the question	Clearly misses the main point

Table 15 presents the end-to-end evaluation results scored by five domain experts. Overall, the expert ratings were relatively higher in Safety and Match, whereas lower scores were mainly observed for several labels in Correctness and Completeness. In particular, Complications, Drug Applicability, Treatment Cost, and Incidence Rate showed relatively weaker performance, indicating that the system still has limitations in handling categories involving greater medical complexity, uncertainty, or individual variation. In contrast, Disease Definition, Disease Causes, Department, and Contraindications achieved relatively better results, suggesting that the system performs more reliably on well-defined and knowledge-based medical questions.

**Table 15:** Expert evaluation results for each label in the end-to-end user evaluation.

Label ID	Correctness ± SD	Completeness ± SD	Safety ± SD	Match ± SD
1	1.87 ± 0.35	1.67 ± 0.49	1.93 ± 0.26	2.00 ± 0.00
2	1.93 ± 0.26	1.47 ± 0.52	2.00 ± 0.00	1.93 ± 0.26
3	1.67 ± 0.49	1.53 ± 0.52	2.00 ± 0.00	1.80 ± 0.41
4	1.13 ± 0.52	1.27 ± 0.46	1.93 ± 0.26	1.93 ± 0.26
5	1.20 ± 0.41	1.20 ± 0.41	2.00 ± 0.00	1.80 ± 0.41
6	1.53 ± 0.64	1.40 ± 0.51	1.93 ± 0.26	1.93 ± 0.26
7	1.67 ± 0.62	1.53 ± 0.64	1.93 ± 0.26	2.00 ± 0.00
8	1.67 ± 0.49	1.40 ± 0.63	1.80 ± 0.41	1.93 ± 0.26
9	1.93 ± 0.26	1.73 ± 0.46	2.00 ± 0.00	2.00 ± 0.00
10	1.47 ± 0.52	1.33 ± 0.82	1.93 ± 0.26	1.93 ± 0.26
11	1.73 ± 0.46	1.40 ± 0.63	2.00 ± 0.00	1.93 ± 0.26
12	1.00 ± 0.65	0.87 ± 0.74	1.80 ± 0.41	1.93 ± 0.26
13	1.07 ± 0.46	1.07 ± 0.26	2.00 ± 0.00	1.80 ± 0.41
14	0.80 ± 0.41	1.00 ± 0.65	1.80 ± 0.41	1.80 ± 0.41
15	1.67 ± 0.49	1.40 ± 0.74	1.93 ± 0.26	1.93 ± 0.26
16	1.40 ± 0.83	0.87 ± 0.74	1.60 ± 0.83	1.80 ± 0.41

Table 16 summarizes the end-to-end evaluation results scored by 12 general participants. Compared with the expert ratings, the user scores showed a similar overall trend, with relatively lower results still concentrated in several more difficult labels. Specifically, Symptoms Check, Treatment Methods, Prevention, Drug Applicability, and Treatment Cost received relatively lower scores. In contrast, Disease Definition, Disease Causes, Disease Symptoms, Contraindications, and Inspection Plan obtained relatively higher scores.

**Table 16:** General participant evaluation results for each label in the end-to-end user evaluation.

Label ID	Correctness ± SD	Completeness ± SD	Safety ± SD	Match ± SD
1	1.92 ± 0.28	1.64 ± 0.49	1.75 ± 0.44	1.89 ± 0.32
2	1.89 ± 0.32	1.53 ± 0.51	1.92 ± 0.28	1.75 ± 0.44
3	1.78 ± 0.42	1.67 ± 0.48	1.75 ± 0.44	1.89 ± 0.32
4	1.17 ± 0.45	0.89 ± 0.71	1.28 ± 0.51	1.25 ± 0.50
5	1.19 ± 0.47	1.25 ± 0.60	1.39 ± 0.49	1.69 ± 0.47
6	1.39 ± 0.55	1.28 ± 0.66	1.53 ± 0.56	1.64 ± 0.49
7	1.50 ± 0.56	1.33 ± 0.53	1.50 ± 0.56	1.67 ± 0.48
8	1.25 ± 0.50	1.28 ± 0.51	1.42 ± 0.50	1.67 ± 0.48
9	1.67 ± 0.48	1.44 ± 0.50	1.89 ± 0.32	1.75 ± 0.44
10	1.75 ± 0.44	1.42 ± 0.50	1.92 ± 0.28	1.86 ± 0.35
11	1.72 ± 0.45	1.53 ± 0.56	1.86 ± 0.35	1.81 ± 0.40
12	1.67 ± 0.53	1.31 ± 0.52	1.78 ± 0.42	1.78 ± 0.42
13	1.33 ± 0.53	1.25 ± 0.55	1.75 ± 0.44	1.69 ± 0.47
14	1.25 ± 0.65	1.14 ± 0.59	1.56 ± 0.50	1.75 ± 0.44
15	1.72 ± 0.45	1.44 ± 0.50	1.81 ± 0.40	1.67 ± 0.48
16	1.39 ± 0.49	1.69 ± 0.52	1.83 ± 0.38	1.56 ± 0.50

Table 17 presents the combined end-to-end evaluation results by integrating the scores from domain experts and general participants, with the total scores further converted into normalized percentages for more intuitive comparison. In Table 17, the values outside parentheses represent the scores from domain experts, whereas the values inside parentheses represent the scores from general participants. The table also reports the 95% confidence interval results of the combined scores, which remain within a relatively reasonable range overall. The combined results show that the overall pattern was broadly consistent between expert and public evaluations: labels such as Contraindications, Disease Definition, Disease Causes, and Department remained among the best-performing categories, whereas Treatment Cost, Complications, Drug Applicability, and Incidence Rate consistently ranked lower. At the same time, general participants tended to assign relatively more favorable scores to common and easy-to-understand categories, while expert ratings were more sensitive to deficiencies in professional accuracy and completeness. Overall, the comparison between the two groups suggests that the proposed system is generally usable. However, further efforts are still needed to improve its performance on labels involving greater uncertainty, stronger personalization, and higher medical complexity.

**Table 17:** Combined end-to-end evaluation results by label.

Label_id	Mean ± SD	95% CI	Normalized score
1	7.47 ± 0.83 (7.19 ± 0.63)	7.00–7.93 (6.84–7.55)	93.33 (89.93)
2	7.33 ± 0.49 (7.08 ± 0.51)	7.06–7.60 (6.79–7.37)	91.67 (88.54)
3	7.00 ± 0.53 (7.08 ± 0.45)	6.70–7.30 (6.83–7.34)	87.50 (88.54)
4	6.27 ± 0.96 (4.58 ± 1.23)	5.73–6.80 (3.89–5.28)	78.33 (57.29)
5	6.20 ± 0.86 (5.53 ± 0.74)	5.72–6.68 (5.11–5.95)	77.50 (69.10)
6	6.80 ± 1.21 (5.83 ± 0.87)	6.13–7.47 (5.34–6.33)	85.00 (72.92)
7	7.13 ± 1.13 (6.00 ± 0.57)	6.51–7.76 (5.68–6.32)	89.17 (75.00)
8	6.80 ± 1.21 (5.61 ± 0.57)	6.13–7.47 (5.29–5.93)	85.00 (70.14)
9	7.67 ± 0.62 (6.75 ± 0.73)	7.32–8.01 (6.34–7.16)	95.83 (84.37)
10	6.67 ± 1.29 (6.94 ± 0.55)	5.95–7.38 (6.53–7.34)	83.33 (86.81)
11	7.07 ± 0.96 (6.92 ± 0.74)	6.53–7.60 (6.50–7.34)	88.33 (86.46)
12	5.60 ± 1.24 (6.53 ± 0.81)	4.91–6.29 (6.07–6.99)	70.00 (81.60)
13	5.93 ± 0.59 (6.03 ± 0.86)	5.60–6.26 (5.54–6.51)	74.17 (75.35)
14	5.40 ± 0.51 (6.69 ± 1.11)	5.12–5.68 (6.06–7.32)	67.50 (71.88)
15	6.93 ± 1.63 (6.64 ± 0.54)	6.29–7.58 (6.33–6.94)	86.67 (82.99)
16	5.67 ± 1.91 (6.47 ± 0.73)	4.61–6.73 (6.06–6.89)	70.83 (80.90)

## 5. Discussion

In Experiment 1, the proposed model consistently outperformed the baseline across multiple evaluation metrics on MTCD, MTCD-n, Weibo, and Toutiao. These results suggest that the model is effective not only on the self-constructed medical dataset but also on public datasets with different label structures and linguistic styles, indicating good robustness and transferability in text intent recognition tasks.

In Experiment 2, analysis of the large model outputs suggests that their relatively low precision is mainly associated with over-identification. For example, in the sentence “What should I do if medication doesn’t work for a child over one year old with a cough?”, the gold entities are {child (crowd), cough (symptom)}, whereas the model additionally identified “over one year old”, “medication”, and “ineffective”. This example reflects the model’s tendency to extract a broader set of potentially relevant spans. Such over-identification may partly arise from missing labels, ambiguous entity boundaries, or annotation inconsistencies in the dataset, which are difficult to eliminate completely in large-scale medical annotation. Therefore, not all additional predictions should be interpreted as equally severe errors. However, this phenomenon should still be treated cautiously in medical question-answering scenarios. Although broader extraction may help reduce missed entities during retrieval, it may also introduce irrelevant or even misleading evidence into downstream answer construction. Combined with the results in Tables 9 and 11, these findings suggest that large language models currently show potential for improving recall and entity coverage, but they still lag behind the strongest traditional baselines in precision and overall F1, and thus cannot yet be concluded to be more practical for medical QA.

In Experiment 3, further analysis of user question-answering records suggests that the relatively low scores of several question types, as discussed in Section 4.4, may be attributed to two main factors. First, questions related to cure rates, treatment costs, treatment duration, and incidence rates are inherently uncertain and often depend on individual conditions, disease stages, regional differences, and clinical contexts. Second, the knowledge graph currently contains relatively limited information for these categories, which makes it difficult for the large language model to generate sufficiently complete answers when only short supplementary descriptions are available. These findings indicate a limitation of the current system in handling question types that require richer contextual information and higher knowledge coverage. In addition, the multimodal knowledge graph in the current study mainly serves to associate medical images with graph nodes and present them in the QA interface. Its contribution to retrieval quality, reasoning quality, or final answer accuracy has not yet been quantitatively validated through dedicated controlled experiments, which remains a common challenge in the evaluation of complex multi-stage systems [40]. Future work will therefore explore more targeted response strategies for such categories, including expanding knowledge support, improving answer-generation control, adaptively adjusting response length according to question type, and further evaluating the specific contribution of image information through controlled comparative studies.

## 6. Conclusion

This paper addresses several key challenges in medical question-answering systems, including intent recognition accuracy, entity recognition under limited labeled data, and the integrated organization and presentation of image-associated medical knowledge. For intent recognition, the proposed dual-layer attention text classification model significantly enhances the capture of long-range textual dependencies and global semantics by introducing a global context attention mechanism. Ablation experiments on the self-built MTCD dataset and public datasets demonstrate that this model effectively mitigates performance degradation in multi-label classification, exhibiting strong generalization capabilities. For entity recognition, we explore an alternative approach based on large language models. By standardizing entity definitions and designing precise instructions, we achieve zero-shot/few-shot entity recognition aligned with knowledge graph nodes. Experiments reveal that while large models exhibit over-identification and therefore lower precision, their relatively high recall suggests potential value for retrieval-oriented medical QA. However, their lower precision and overall F1 indicate that they should currently be regarded as a promising low-resource alternative rather than a replacement for stronger conventional NER baselines. Regarding system integration and visualization, we constructed a multimodal medical knowledge graph and implemented an integrated retrieval and front-end visualization framework using Flask and ECharts. The current results support its utility for multimodal knowledge organization and display, while the specific contribution of image information to retrieval quality and answer quality still requires further controlled validation. End-to-end evaluations indicate that the system is generally effective for well-defined knowledge-based medical questions, while weaker performance remains for categories involving greater uncertainty, stronger personalization, and limited knowledge coverage. Future work should focus on improving support for difficult question categories, expanding knowledge coverage for weakly supported labels, refining answer-generation control for uncertain and personalized questions, and conducting controlled studies to quantify the contribution of multimodal information to retrieval and answer quality.
